# Community-Wide Health Risk Assessment Using Geographically Resolved Demographic Data: A Synthetic Population Approach

**DOI:** 10.1371/journal.pone.0087144

**Published:** 2014-01-28

**Authors:** Jonathan I. Levy, Maria Patricia Fabian, Junenette L. Peters

**Affiliations:** Department of Environmental Health, Boston University School of Public Health, Boston, Massachusetts, United States of America; National Taiwan University, Taiwan

## Abstract

**Background:**

Evaluating environmental health risks in communities requires models characterizing geographic and demographic patterns of exposure to multiple stressors. These exposure models can be constructed from multivariable regression analyses using individual-level predictors (microdata), but these microdata are not typically available with sufficient geographic resolution for community risk analyses given privacy concerns.

**Methods:**

We developed synthetic geographically-resolved microdata for a low-income community (New Bedford, Massachusetts) facing multiple environmental stressors. We first applied probabilistic reweighting using simulated annealing to data from the 2006–2010 American Community Survey, combining 9,135 microdata samples from the New Bedford area with census tract-level constraints for individual and household characteristics. We then evaluated the synthetic microdata using goodness-of-fit tests and by examining spatial patterns of microdata fields not used as constraints. As a demonstration, we developed a multivariable regression model predicting smoking behavior as a function of individual-level microdata fields using New Bedford-specific data from the 2006–2010 Behavioral Risk Factor Surveillance System, linking this model with the synthetic microdata to predict demographic and geographic smoking patterns in New Bedford.

**Results:**

Our simulation produced microdata representing all 94,944 individuals living in New Bedford in 2006–2010. Variables in the synthetic population matched the constraints well at the census tract level (e.g., ancestry, gender, age, education, household income) and reproduced the census-derived spatial patterns of non-constraint microdata. Smoking in New Bedford was significantly associated with numerous demographic variables found in the microdata, with estimated tract-level smoking rates varying from 20% (95% CI: 17%, 22%) to 37% (95% CI: 30%, 45%).

**Conclusions:**

We used simulation methods to create geographically-resolved individual-level microdata that can be used in community-wide exposure and risk assessment studies. This approach provides insights regarding community-scale exposure and vulnerability patterns, valuable in settings where policy can be informed by characterization of multi-stressor exposures and health risks at high resolution.

## Introduction

Evaluation of environmental health risks in communities has increasingly focused on the combined risks to health from multiple agents or stressors, defined by the United States Environmental Protection Agency (US EPA) as “cumulative risk assessment” [1]. This is in part because of the growing recognition that background exposures and susceptibility characteristics need to be considered in developing appropriate dose-response models, but also relates to environmental justice concerns and general decision relevance [2], [3]. This cumulative risk framework theoretically considers not only multiple chemical exposures, but also the effects of social stressors and other factors of the built and social environment that operate at either individual or community levels [4].

While this framework is appealing, there are significant analytical challenges. One such challenge is developing exposure models, which must have sufficient geographic and demographic resolution to provide relevant contrasts within the community and to identify high-risk subpopulations given correlations (positive or negative) among stressors.

It is impractical to gather detailed exposure measurements or exposure-related information from all members of a community, so in many cases investigators must leverage a limited number of exposure measurements and develop broadly applicable exposure models. For air pollutants of ambient origin, monitoring networks and public geospatial databases may be sufficient to evaluate exposures, but for most other stressors, additional information would be required. For example, mercury exposure is typically dominated by fish consumption, which may be influenced by income, race/ethnicity, and other factors [5]. Smoking has been predicted by gender, age, income, race/ethnicity, education, occupation, household configuration, and community-level factors such as poverty rate and the presence of a state cigarette tax [6]. Cord blood polychlorinated biphenyl (PCB) levels have been related to factors such as maternal age, country of origin, and dietary patterns [7]. Blood lead has been associated with income, race, and age of home [8]. A common feature of these models is that they use multivariable regression to explain exposure variability, generally using predictors that could be obtained from public databases or approximated using standard assumptions.

A significant barrier in applying these models to specific communities is the lack of geographically resolved multivariable data on key predictors. For example, the US Census provides the distribution of home age and the distribution of household income at the census tract level, but not the cross-tabulated distribution of home age by household income. If attributes are correlated with one another, which is generally the case given strong socioeconomic interdependence for many attributes, the lack of this information will lead to erroneous exposure models. Census microdata provide the detailed individual-level data needed to determine these interdependencies, but only at very coarse geographic resolution, because of the need to limit potential identifiability. Most exposure regression models involve a blend of geographically resolved (i.e., proximity to sources, home attributes) and demographically resolved (i.e., age by income by race/ethnicity) predictors, but it is not possible to obtain multivariable demographic information with high-resolution geographic data from public data resources.

Simulation approaches can be used to construct geographically resolved microdata, using optimization techniques to maximize the fit between individual-level microdata lacking geographic resolution and aggregated census data at high geographic resolution [9], [10]. Synthetic microdata have been developed for a variety of applications, including transportation planning [11], micro-marketing [12], and health behavior modeling [13]. However, to our knowledge, these methods have never been used for community-scale modeling of environmental health risks.

In this paper, we develop synthetic microdata for a low-income community living near a Superfund site (New Bedford, Massachusetts), where these synthetic microdata include multivariable individual-level attributes and spatially resolved geographic assignment. We then constructed a new model of the individual-level likelihood of smoking in New Bedford as a function of the microdata characteristics. We linked this model to the synthetic microdata as a demonstration of the usefulness of developing synthetic microdata in providing insight about the geographic and demographic patterns of exposure to a key risk factor for the residents of New Bedford, which can have further implications for use in characterizing multi-stressor exposures.

## Methods

### Location

**Figure 1:**
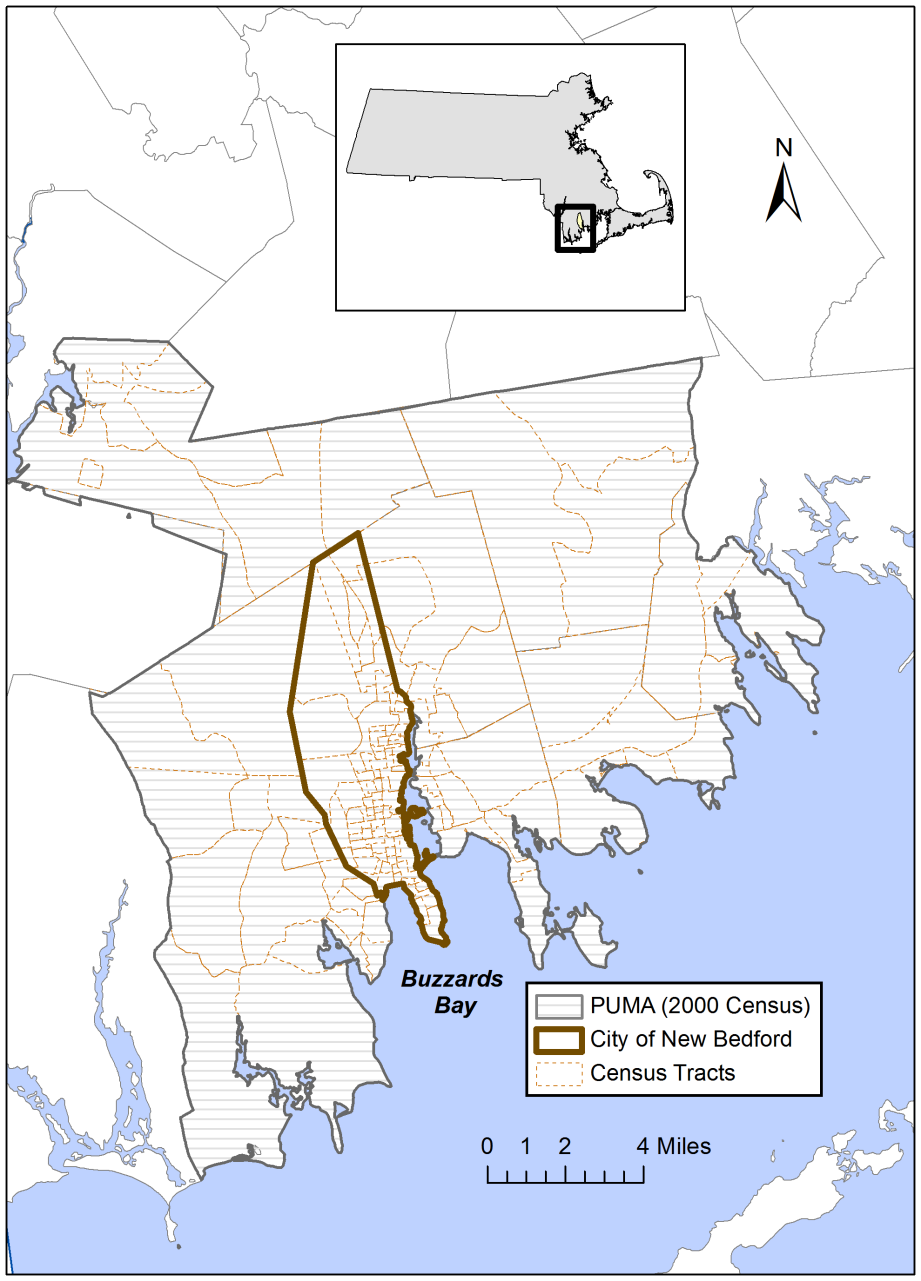
Map of the New Bedford study area.

New Bedford, Massachusetts is a diverse urban city with approximately 95,000 residents in 2010 (Figure 1). The New Bedford Harbor was designated a Superfund site in 1982, related to PCB contamination, and New Bedford contains a number of hazardous waste sites, industrial sources, and nearby major roadways [14]. Of the 233 census-designated places in Massachusetts in the 2006–2010 American Community Survey (ACS), New Bedford had the 14^th^-lowest median household income. Residents had elevated rates of adult diabetes, hypertension, high cholesterol, smoking, and obesity [15], [16], as well as elevated rates of low birth weight, inadequate prenatal care, and high childhood blood lead [17]. New Bedford is therefore a highly vulnerable and susceptible community, for which cumulative risk assessments can be informative.

### Simulation methodology

Two general approaches have been used in the literature to generate synthetic microdata [10] - reweighting, in which the existing microdata are assigned sets of weights for each small area to best match the aggregate distributions, and synthetic reconstruction, in which the microdata are probabilistically generated given the aggregate distributions. We focus on reweighting approaches in this analysis, given our interest in characterizing a large number of population characteristics that may predict exposure or vulnerability.

**Figure 2:**
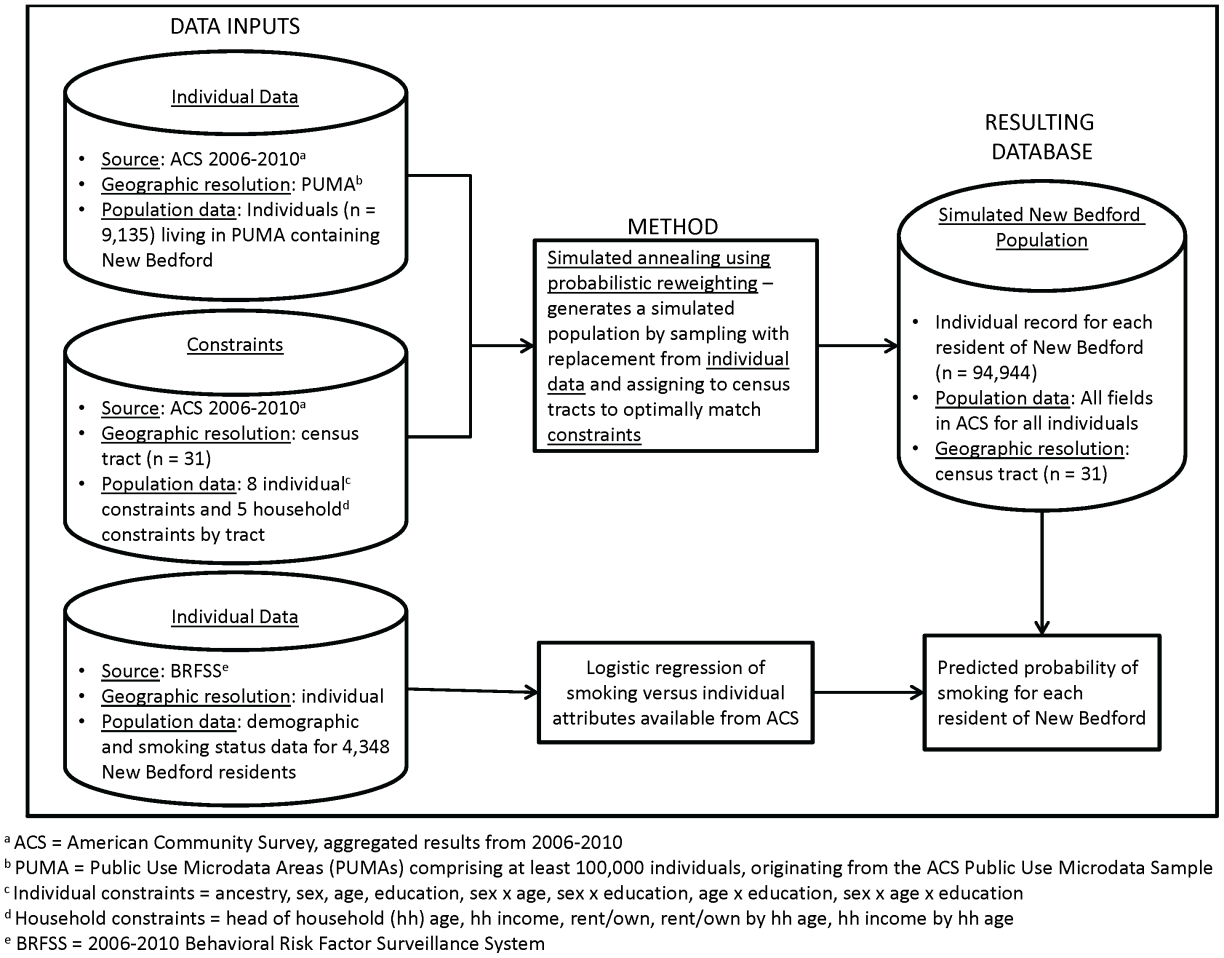
Approach to generate geographically resolved synthetic microdata and estimate geographic and demographic smoking patterns in New Bedford.

Probabilistic reweighting using simulated annealing has been shown to perform slightly better than alternative reweighting approaches, such as deterministic reweighting or conditional probability modeling [9]. As illustrated in Figure 2, this entails selecting a random subset of households from microdata and comparing to aggregate area-level constraints, sequentially replacing individual households to see if the fit improves [18]. The simulated annealing approach helps ensure that global rather than local optima are obtained. We applied this methodology using the software package CO [19], which has been tested in multiple applications and considered to be more robust than alternative algorithms [10].

### Census data

Generating synthetic microdata using probabilistic reweighting requires two basic types of data – multivariable individual-level microdata lacking resolved geographic identifiers, and small area constraints to place the individuals in appropriate locations. We derived both from the ACS 5-year database (2006–2010). The ACS surveys a fraction of the US population each year, and the 5-year estimates are generally used to determine estimates for smaller geographic areas (like census tracts). For the microdata, we used ACS data from the Public Use Microdata Sample (PUMS), which provides geographic identifiers for Public Use Microdata Areas (PUMAs) comprising at least 100,000 individuals. The PUMA of interest for our analysis includes New Bedford as well as bordering or nearby communities (Figure 1). Based on Census 2000 population estimates, 54% of the people living in this PUMA reside in New Bedford. Of note, if the demographics differ significantly between surrounding communities and New Bedford, the simulation approach will preferentially select those microdata surveys consistent with the small-area constraints for New Bedford, reducing potential errors associated with PUMA resolution.

For small area constraints, we focused on the census tract as the unit of analysis, because more candidate constraints were available at the census tract level than at the block group level. There are 31 census tracts in New Bedford (Figure 1), with population by tract ranging from 1,448 to 5,591. Candidate constraints were either associated in the literature with exposures or health outcomes of interest for our cumulative risk assessments, available in cross-tabulations (i.e., age by household income, rather than only having age and household income as separate constraints), or suggested by community partners given insight about neighborhood differences. We included 8 individual-level constraints (ancestry, sex, age, educational attainment, sex by age, sex by educational attainment, age by educational attainment, and sex by age by educational attainment) and 5 household-level constraints (head of household age, household income, rent/own, rent/own by head of household age, household income by head of household age). Ancestry was collapsed into 13 categories representing the main countries of origin in the community and those previously associated with environmental exposures of interest in New Bedford [7]. For all other fields, categories were used as provided in the ACS.

### Optimization approach

For a given set of constraints, the goodness of fit was evaluated using the relative sum of square Z-scores associated with individual constraint tables, in comparison with the chi-square critical value (p < 0.05). As described by Williamson [19], this approach minimizes the proportional differences between constraints and weighted estimates, versus approaches that focus on the absolute differences. We additionally examined the sum of the total absolute errors across constraint tables, which is generally considered to be problematic if the sum exceeds the number of households in a given geographic area (i.e., if total absolute error divided by number of households exceeds 1) [19]. Of note, if a small number of relatively weak constraints are used, it will be easy to match these constraints with limited error, but there may be significant error in the distribution of other microdata fields. We therefore attempted to impose the largest number of constraints possible, without leading to excessive error.

### Microdata evaluation and application

Synthetic microdata need to be evaluated across three major dimensions to ensure that the individuals have been assigned to appropriate geographic areas, therefore making the microdata applicable to community-scale modeling of environmental health risks.

Evaluation 1. First, the degree of error needs to be assessed for the microdata fields included in the constraints (e.g., the total absolute error divided by the number of households).

Evaluation 2. Second, the degree of error needs to be assessed for microdata fields that are not included in the constraints but are potentially important for evaluating environmental health risks in the community. While greater error would be anticipated for fields not included as constraints, it is important to know whether the complete multivariable microdata reasonably reflect the spatial and demographic patterns described in the ACS. For this second evaluation, we focused on whether the geographically resolved microdata reasonably predict the percentage of multi-family homes and number of rooms per home by census tract. Both of these variables are potentially relevant for housing-related exposures and are not simply explained by the constraint variables (i.e., while income and home type are clearly correlated, there is inherent spatial patterning related to zoning and proximity to commercial areas).

Evaluation 3. Third, the synthetic microdata must be able to characterize geographic and demographic distributions for key stressors of interest not available in the microdata, using regression models explaining variability as a function of the microdata. These distributions cannot be directly validated in the same manner as in the first two evaluations above, because the lack of geographically and demographically refined exposure data is the precise rationale for this modeling approach. However, some of the aggregated estimates can be compared with surveillance data or previous models. To conduct the third evaluation, we developed a multivariable regression model for smoking, given that direct smoking and resulting environmental tobacco smoke are associated with multiple health outcomes of concern to the community. We obtained 2006–2010 Behavioral Risk Factor Surveillance System (BRFSS) data from the Massachusetts Department of Public Health (MA DPH), which contains smoking status and extensive demographic data for 4,348 individuals living in New Bedford across the five study years. We constructed a multivariable logistic regression to predict smoking status as a function of demographic variables. We considered as candidate predictors variables that had previously been associated with smoking in the literature and were available from ACS-derived synthetic microdata, including age, gender, marital status, race, ancestry, educational attainment, household income, and employment status. All predictors were constructed to be identical in structure as the covariates in the synthetic microdata, and both the regression model and microdata are at the individual level, allowing for direct model application. While formal validation is not possible, we predicted smoking prevalence for demographic subpopulations described in MA DPH reports [20] and compared our estimates with reported values, noting that minor discrepancies would be expected given differences in base years.

Because our simulation modeling involved multiple uncertainties, we used Monte Carlo analysis to quantify these uncertainties in the predicted geographic and demographic patterns of smoking in New Bedford. We incorporated uncertainty at two levels. First, we included uncertainty in the smoking regression model by randomly sampling coefficient values given estimated means and standard errors, using the variance-covariance matrix to account for correlations among coefficients. This yields an uncertainty distribution of smoking probabilities for each individual. Second, we randomly assigned smoking status to individuals given these probabilities, so that each individual had a binary (yes/no) assignment in each of the 5000 realizations. This allowed us to characterize uncertainties in estimated smoking rates for any geographic or demographic subpopulation of interest.

## Results

### Evaluation 1

Applying all 13 candidate constraints yielded synthetic microdata for New Bedford which perfectly matched all univariate constraints at the census tract level, with a good overall fit across all constraints (Table 1). The last three columns in Table 1 are examples of population characteristics estimated for each census tract, including % Portuguese, % Cape Verdean, and % age above 25 years with less than high school education.

**Table 1:** Population characteristic examples and goodness of fit statistics for census tract level synthetic microdata with 13 constraints simultaneously imposed.

			% population in each census tract for 3 example characteristics		
Census tract	Number of people	Overall total absolute error per household	% Portuguese	% Cape Verdean	% age 25+ < HS graduate
6501.01	5591	0.1	46%	5%	25%
6501.02	4666	0.12	43%	4%	24%
6502.01	3025	0.13	37%	2%	26%
6502.02	1973	0.15	35%	1%	31%
6503	3094	0.11	38%	3%	22%
6504	3420	0.11	48%	7%	43%
6505	3430	0.14	47%	2%	31%
6506	2788	0.17	37%	2%	45%
6507	2235	0.21	27%	10%	50%
6508	3306	0.11	41%	6%	45%
6509	3327	0.12	19%	12%	48%
6510.01	2857	0.24	41%	7%	24%
6510.02	4022	0.11	32%	6%	23%
6511	4053	0.11	22%	27%	34%
6512	1810	0.28	19%	11%	46%
6513	2268	0.25	17%	14%	31%
6514	3440	0.16	23%	14%	35%
6515	3326	0.14	23%	21%	22%
6516	4119	0.13	22%	32%	28%
6517	2235	0.27	16%	25%	38%
6518	1448	0.09	22%	16%	44%
6519	2419	0.4	14%	17%	49%
6520	2821	0.28	53%	10%	41%
6521	2799	0.2	40%	2%	22%
6522	2875	0.2	41%	8%	22%
6523	2916	0.14	48%	5%	42%
6524	2536	0.14	58%	5%	43%
6525	2426	0.19	52%	8%	49%
6526	2790	0.24	27%	16%	53%
6527	3381	0.13	39%	5%	42%
6528	3548	0.15	65%	1%	33%

All population characteristics in the table were identical for the synthetic microdata and the American Community Survey data.

The overall total absolute error per household was well under 1 in all census tracts, exceeding 0.3 in only one census tract. For all seven univariate constraints as well as four multivariable constraints (sex by age, sex by educational attainment, age by educational attainment, and rent/own by head of household age), the relative sum of squared Z-scores never exceeded the chi-square critical value, with zero or near-zero values. Only the two most complex multifactorial constraints with many categories (sex by age by educational attainment and household income by head of household age, with 72 and 65 categories, respectively) displayed enough error to exceed the chi-square critical value for some of the census tracts. Examining the fits for those tables, differences were generally modest and reflected small population numbers in adjacent cells along at least one dimension. When the number of constraints was loosened (i.e., by removing two-way person-level constraints), the degree of error increased across both error metrics. We therefore concluded that application of all 13 constraints yielded the most accurate synthetic microdata.

### Evaluation 2

**Figure 3:**
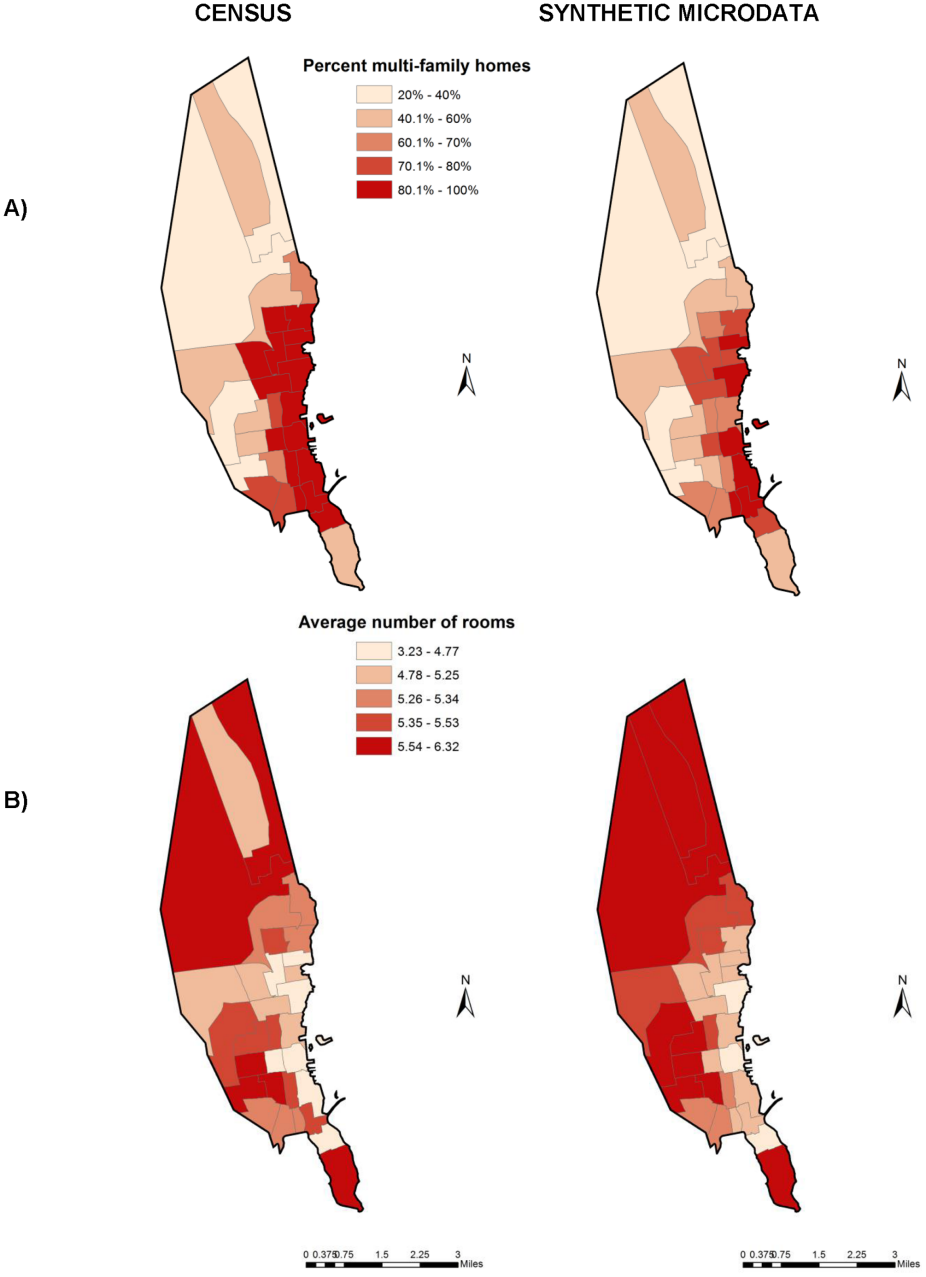
A) Percentage of multi-family homes and B) average number of rooms per household by census tract in New Bedford, as reported by the American Community Survey and estimated by synthetic microdata.

To determine whether the synthetic microdata appropriately assigned individuals to census tracts, we also evaluated the percentage of multi-family homes and average number of rooms per home by census tract, as predicted by the synthetic microdata and as reported in the ACS. As shown in Figure 3, the synthetic microdata reasonably reproduced the spatial patterns of these covariates, with a high correlation by census tract (0.96 for percentage of multi-family homes, 0.91 for average number of rooms per home). Some modest bias existed in the estimated percentage of multi-family homes (69% in the ACS, 61% in our synthetic microdata), potentially related to the inclusion of some suburban households outside of New Bedford in the synthetic microdata. There was no appreciable bias in the number of rooms per home (mean of 5.3 for both datasets).

### Evaluation 3

To illustrate the application of the synthetic microdata, we combined it with a multivariable regression model of smoking to evaluate the resulting demographic and geographic patterns of smoking for New Bedford. In our multivariable logistic regression of smoking, all tested covariates were statistically significant (Table 2). Smoking rates were elevated among individuals who were male, non-Hispanic, without a college education, unemployed or out of the labor force, with lower household income, unmarried, and working age. Age and educational attainment were the two strongest predictors.

**Table 2:** Multivariable logistic regression model of smoking as a function of demographic characteristics in New Bedford.

Covariate	Beta	Standard error	p-value
Intercept	–5.1	0.65	< 0.0001
Male	0.27	0.089	0.0022
Race/ancestry			
White non-Hispanic	0.35	0.13	0.0095
Black non-Hispanic	0.27	0.21	0.20
Mexican	–0.56	0.23	0.013
Dominican	–0.71	0.45	0.11
Puerto Rican	–0.72	0.69	0.30
Educational attainment			
< 8^th^ grade	0.36	0.19	0.056
9^th^–11^th^ grade	1.1	0.17	< 0.0001
High school graduate	0.68	0.13	< 0.0001
Some college	0.49	0.14	0.0003
Employment status			
Employed	–0.30	0.11	0.0068
Unemployed	0.14	0.15	0.35
Household income			
< 10,000	0.97	0.21	< 0.0001
10,000–14,999	0.89	0.22	< 0.0001
15,000–19,999	0.76	0.20	0.0001
20,000–24,999	0.69	0.19	0.0003
25,000–34,999	0.67	0.19	0.0003
35,000–49,999	0.50	0.17	0.0041
50,000–74,999	0.39	0.18	0.027
Marital status			
Married	–0.48	0.12	< 0.0001
Divorced	0.047	0.13	0.71
Widowed	–0.21	0.18	0.24
Separated	–0.060	0.20	0.76
Age			
18–24	2.9	0.63	< 0.0001
25–29	3.6	0.62	< 0.0001
30–34	3.5	0.62	< 0.0001
35–39	3.5	0.62	< 0.0001
40–44	3.4	0.62	< 0.0001
45–49	3.7	0.61	< 0.0001
50–54	3.4	0.61	< 0.0001
55–59	3.1	0.61	< 0.0001
60–64	2.9	0.61	< 0.0001
65–69	2.3	0.61	0.0001
70–74	1.8	0.62	0.0038
75–79	1.4	0.64	0.027
80–84	0.79	0.69	0.25

References: female, other race/ancestry, college graduate, not in labor force, household income > =  $75,000, never married or unmarried couple, age 85+.

**Figure 4:**
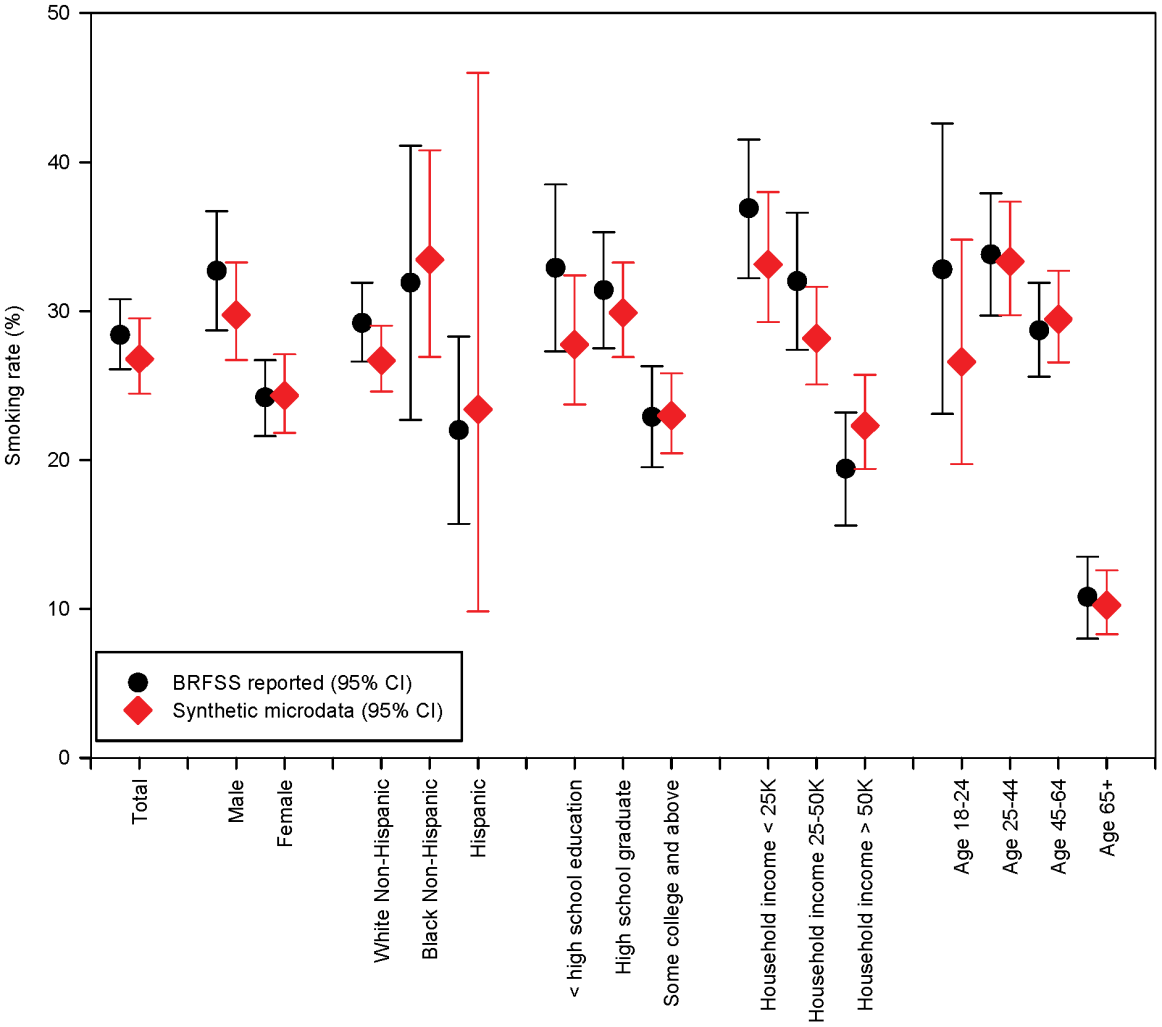
Smoking rate by demographic group in New Bedford, as reported by the BRFSS and estimated using synthetic microdata.

**Figure 5:**
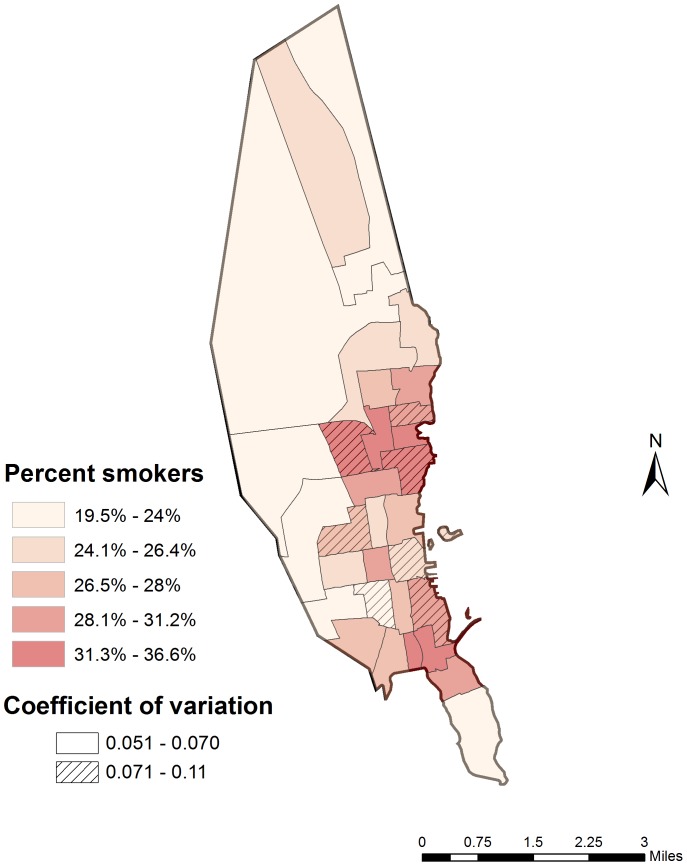
Smoking rate by census tract in New Bedford, estimated using synthetic microdata.

Applying this multivariable regression model to the synthetic microdata, there was a wide range of predicted probabilities for individuals, consistent with strong demographic patterning of smoking behaviors – about 8% of the population had a mean smoking probability above 50%, while 14% of the population had a mean smoking probability under 10%. The smoking rates for key demographic groups corresponded closely with reported surveillance data as derived from the BRFSS (Figure 4), in spite of the fact that the two sets of estimates are based on slightly different underlying data. The linkage of this regression model with the synthetic microdata also allowed us to estimate smoking rates by census tract in New Bedford (Figure 5). Given demographic patterns that vary across the city, the estimated smoking rates by census tract ranged from a low of 20% (95% CI: 17%, 22%) to a high of 37% (95% CI: 30%, 45%).

## Discussion

Using simulation methods, we constructed geographically resolved synthetic microdata for a high-risk community and demonstrated its utility for predicting geographic and demographic patterns of smoking. The individual-level microdata were statistically robust, with only minor misrepresentation of complex cross-tabulated constraints at census tract resolution. Sensitivity analyses showed that removing those constraints led to poorer overall model performance, indicating the value of the cross-tabulated constraints in providing a more accurate characterization of the individual constraint variables. We were able to connect these microdata with a strong predictive model of smoking behavior as a function of multivariable demographic characteristics, relying on local data to ensure that demographic variables retain their local context. For example, general population smoking regression models [6] would have greatly misrepresented smoking rates in New Bedford, given some of its unique demographics (e.g., large Portuguese and Cape Verdean populations) and its status as a city with high smoking rates located in a state with low smoking rates. Our resulting small area estimates of smoking prevalence are consistent with modeled patterns in other studies [13], [21], with significant spatial heterogeneity based on underlying demographic patterns.

The multivariable demographic information and corresponding regression models predicting exposures would allow us to rapidly identify high-risk subpopulations for specific health outcomes. As a simple example, previously-derived multivariable regression models constructed from New Bedford data [7] showed that cord serum PCB levels are elevated among Cape Verdean women over 35 years of age. Our geographically resolved synthetic microdata can be used to rapidly determine that approximately 0.9% of New Bedford’s adult population consists of Cape Verdean women over 35 (but of childbearing age), with more than half of these individuals clustered in five census tracts. Moreover, maternal smoking has some cardiovascular and neurodevelopmental health effects in common with PCBs, and smoking status may also predict PCB levels. Our synthetic microdata allow us to determine that the lower-income subset of this population would be at particularly high risk given elevated smoking rates, with three of the five census tracts displaying elevated predicted smoking rates among Cape Verdean women over 35. While this is a somewhat stylized example and prioritization would require formal characterization of exposures and cumulative risks to multiple stressors, this example illustrates how our synthetic microdata could allow for targeted public health outreach and public policy development.

In spite of the strong model performance and potential generalizability of our modeling strategy, there are some limitations in our synthetic microdata and corresponding smoking models, as well as in the general approach. While ACS microdata include a large number of covariates, there remain gaps in the individual-level information available, and the synthetic microdata are ultimately built on a population sub-sample that may contribute uncertainties in characterizing multiple exposures for smaller (community-relevant) areas. With an average of 3,063 residents per census tract in New Bedford (range: 1,448 – 5,591), the 5% sample of the population available in the ACS microdata will not be able to fully capture all multivariable demographic nuances. In addition, ACS microdata will underrepresent undocumented populations that may be at particular risk.

Our modeling approach is also predicated on the assumption that basic demographic characteristics are strong predictors of environmental exposures. While this is clearly the case for smoking, it may not hold more broadly. For example, for pollutants with strong spatial gradients, we may not adequately capture exposure contrasts using census tract resolution. However, there is nothing in our analytical approach that requires census tract resolution, and relying on individual-level data as the foundation for our modeling affords flexibility for future applications. It may also be challenging to communicate our models and findings to communities or policymakers, as the structure of the models suggests that exposures are dictated by demographics rather than being a function of complex behaviors and exposure pathways.

Relatedly, there are a limited number of national-scale environmental exposure studies with sufficient geographic resolution, so an analysis that extended beyond smoking or other survey-derived behaviors in the BRFSS would need to rely on some amount of local sampling or surveying. We focused on New Bedford in part because a long-standing pregnancy cohort study [7], [22], [23], [24] includes extensive biomarker data from which we can develop predictive regression models as a function of variables available in the synthetic microdata, but such data are not available in all communities.

In spite of these limitations, our modeling approach offers some significant advantages over other options. Geographically resolved synthetic microdata provide community-scale detail that would rarely be available for cumulative risk assessment models, including exposure predictors and vulnerability attributes. By building both the microdata and the smoking regression model at the individual level, we yield robust spatial estimates and avoid issues such as the modifiable areal unit problem. Subpopulations at high risk across multiple exposures can be identified and targeted for public health interventions. Our strategy would also allow for any exposure characterization study to rapidly yield community-scale conclusions, provided that the requisite demographic and geographic data were collected from study participants.

## Conclusions

Our simulation approach provides a method to create detailed and geographically resolved individual microdata for a low-income population that can be used in community-wide environmental exposure and risk assessment studies. We applied the method to a regression model of smoking behavior to illustrate the applicability of our approach. While our population characteristics and resulting models were specific to New Bedford, all our methods and input data generalize to any other city in the US, providing a powerful mechanism to compare health risk factors within and between major cities, and allowing for more targeted public health interventions. In future studies, these microdata will be connected with comparable regression models for multiple environmental exposures to provide detailed characterization of exposure patterns relevant to health outcomes of interest in New Bedford.
